# Supplementary material for: OsMGT1 Confers Resistance to Magnesium Deficiency By Enhancing the Import of Mg in Rice
Source: Int J Mol Sci. 2019 Jan 8;20(1):207. doi: 10.3390/ijms20010207 (PMC6337559; doi:10.3390/ijms20010207)
Supplement: Supplementary file 1 [file ijms-20-00207-s001.pdf]

Supplemental Table 1. Primers used for vector construction and Real Time PCR

Primer	Forward primer	Reverse primer
Ubi: OsMGT1	TAGGTACCATGGAGCGGAGGGCGCAGCC	CGGGATCCTCACTGCAGGATCTTGCTCT
OsMGT1-RT	CAAGTACGCATCTGTTGATC	CGCGTATTCACGGATATGGTACAGGG
OsMGT2-RT	AAGTAGGCTAGTTGCTCTGA	AGAACCATAGGAATCCGCAG
OsMGT3-RT	CAAGCATTGGTTGAGCTGGG	GGAACGGAGCACTCCATGTT
OsMGT4-RT	TGCACCTAACGGAGAAGCTC	TGTCCTCCACGTACTCCCTCA
OsMGT5-RT	GCAGAATTTACTGATATGGA	CAGCTCATCAAGTGCCGGAT
OsMGT6-RT	CAGGTGCTGGAGCTCGCACT	GTCTTCCACGTCATTATCCA
OsMGT7-RT	AGGTACGTCGCTGAGCTGCA	AGGATAAGCGTCAGCCTCCA
OsMGT8-RT	GACGATGACATGGCTGATCT	CAATTTGCATGAAGTATGCC
OsMGT9-RT	GGAATCCAGGCGATGCCGTT	TTCCAGCACCAGCACCTCAT
